# Distinctive G Protein-Dependent Signaling by Protease-Activated Receptor 2 (PAR2) in Smooth Muscle: Feedback Inhibition of RhoA by cAMP-Independent PKA

**DOI:** 10.1371/journal.pone.0066743

**Published:** 2013-06-18

**Authors:** Wimolpak Sriwai, Sunila Mahavadi, Othman Al-Shboul, John R. Grider, Karnam S. Murthy

**Affiliations:** Department of Physiology, VCU Program in Enteric Neuromuscular Sciences, Virginia Commonwealth University, Richmond, Virginia, United States of America; University of Illinois at Chicago, United States of America

## Abstract

We examined expression of protease-activated receptors 2 (PAR2) and characterized their signaling pathways in rabbit gastric muscle cells. The PAR2 activating peptide SLIGRL (PAR2-AP) stimulated G_q_, G_13_, G_i1_, PI hydrolysis, and Rho kinase activity, and inhibited cAMP formation. Stimulation of PI hydrolysis was partly inhibited in cells expressing PAR2 siRNA, Ga_q_ or Ga_i_ minigene and in cells treated with pertussis toxin, and augmented by expression of dominant negative *r*egulator of *G* protein *s*ignaling (RGS4(N88S)). Stimulation of Rho kinase activity was abolished by PAR-2 or Ga_13_ siRNA, and by Ga_13_ minigene. PAR2-AP induced a biphasic contraction; initial contraction was selectively blocked by the inhibitor of PI hydrolysis (U73122) or MLC kinase (ML-9), whereas sustained contraction was selectively blocked by the Rho kinase inhibitor (Y27632). PAR2-AP induced phosphorylation of MLC_20_, MYPT1 but not CPI-17. PAR2-AP also caused a decrease in the association of NF-kB and PKA catalytic subunit: the effect of PAR2-AP was blocked by PAR2 siRNA or phosphorylation-deficient RhoA (RhoA(S188A)). PAR2-AP-induced degradation of IkBa and activation of NF-kB were abolished by the blockade of RhoA activity by *Clostridium botulinum* C3 exoenzyme suggesting RhoA-dependent activation of NF-kB. PAR2-AP-stimulated Rho kinase activity was significantly augmented by the inhibitors of PKA (myristoylated PKI), IKK2 (IKKIV) or NF-kB (MG132), and in cells expressing dominant negative mutants of IKK (IKK(K44A), IkBa (IkBa (S32A/S36A)) or RhoA(S188A), suggesting feedback inhibition of Rho kinase activity via PKA derived from NF-kB pathway. PAR2-AP induced phosphorylation of RhoA and the phosphorylation was attenuated in cells expressing phosphorylation-deficient RhoA(S188A). Our results identified signaling pathways activated by PAR2 to mediate smooth muscle contraction and a novel pathway for feedback inhibition of PAR2-stimulated RhoA. The pathway involves activation of the NF-kB to release catalytic subunit of PKA from its binding to IkBa and phosphorylation of RhoA at Ser^188^.

## Introduction

Protease-activated receptors (PARs) comprise a family of G protein-coupled receptors with a unique activation mechanism involving proteolytic cleavage of the extracellular N-terminus domain of the receptor to expose a new built-in N-terminus part of the receptor that acts as a ligand (also known as “tethered ligand”). Molecular cloning studies have identified four PARs and these are activated by a large number of proteases [1]. Physiologically thrombin activates PAR1, PAR3 and PAR4, whereas trypsin activates PAR2 [1]–[3]. Each PAR has a unique N-terminal tethered ligand sequence and binding of tethered ligand to the extracellular loop of the receptor results in conformational changes that permit interaction of receptors with heterotrimeric G proteins and leads to activation of a substantial network of signaling pathways. Receptor-specific, synthetic peptides as short as 5–6 amino acids, corresponding to the amino acid sequence of the exposed tethered ligand, known as PAR-activating peptides (PAR-AP) mimic the effect of the proteases independent of the proteolytic cleavage of the receptor [4].

PARs are located in several cell types and play an important role in many physiological functions. The gastrointestinal (GI) tract, of all the body systems, is exposed to the widest array of proteases in both normal situations and during diseases [1], [3], [5]–[7]. PARs, especially PAR1 and PAR2 are abundantly expressed throughout the GI system [7]. PAR2 which is activated by trypsin, tryptase, and other endogenous and exogenous proteases play an important role in several gastrointestinal functions [5], [6], [8]–[11]. PAR2s are present in spinal sensory afferents co-localized with neuropeptides, substance P and calcitonin gene-related peptide and activation of PARs causes release of these neuropeptides, suggesting a role in nociception [12]. PAR2s are also expressed within both excitatory and inhibitory motor neurons suggesting a role in neuronal transmission to regulate GI function such as mucosal protection, secretion and motility [8].

The role of PAR2 in GI motility is complex, and varies with species and tissue. In vivo studies have demonstrated that activation of PAR2 enhances GI transit [13]. In longitudinal strips of mouse gastric fundus, activation of PAR2 causes biphasic responses, relaxation followed by contraction [14], whereas PAR2 activation in rat duodenal longitudinal muscle causes only a small contraction [15]. In colon the effects of PAR2 on circular and longitudinal muscle are distinct: a concentration-dependent reduction of the spontaneous phasic contraction in the circular muscle and contractile effects in the longitudinal muscle [16]. Thus, the effect of PAR activation on gut motility is diverse, which include relaxation, contraction, or biphasic response of relaxation followed by contraction and this could be dependent on whether the activated receptor is present predominantly on smooth muscle cells or enteric neurons. Transmitters released from the enteric neurons, or release of endogenous prostanoids in response to PAR activation, in turn, modulate the intrinsic electrical and mechanical activities of the smooth muscle. Expression of PAR2 receptors and the mechanism underlying their effects on smooth muscle cells of the gastrointestinal tract are not known.

The present study focused on characterizing expression of PAR2 and the signaling pathways to which these receptors are coupled in freshly dispersed and cultured smooth muscle cells of rabbit stomach. The small synthetic peptide SLIGRL, corresponding to tethered ligand sequences, was used to selectively activate PAR2 and to identify the signaling pathways activated by PAR2. Our results demonstrate that PAR2 are coupled to G_q_, G_i_ and G_13_, and stimulation of PI hydrolysis and RhoA/Rho kinase activity to induce muscle contraction. A novel cAMP-independent PKA pathway for feedback inhibition of PAR2-stimulated RhoA was demonstrated involving activation of NF-kB and release of PKA from its binding to IkBa complex leading to phosphorylation of RhoA at Ser^188^.

## Materials and Methods

PAR2-activating peptide (PAR2-AP, SLIGRL) was obtained from Bachem, Torrance, CA; [^125^I]cAMP, [γ-^32^P]ATP, [^32^P]Pi, [^35^S]GTPγS, and [^3^H]myo-inositol were obtained from Perkin Elmer Life Sciences, Boston, MA; Collagenase CLS type II and soybean trypsin inhibitor were obtained from Worthington, Freehold, NJ; Western blotting, Dowex AG-1×8 resin (100–200 mesh in formate form), chromatography material and protein assay kit from Bio-Rad Laboratories, Hercules, CA; antibodies to Ga_q_, Ga_i1_, Ga_i2_, Ga_i3_, Ga_12_, Ga_13_, Ga_s_, phospho-specific MYPT1 (Thr^696^), MLC_20_ (Ser^19^) and CPI-17 (Thr^38^), Rho kinase, PKA catalytic subunit, IKK2(ser^177/181^), IkBa, and p65 subunit were from Santa Cruz Biotechnology, Santa Cruz, CA; myelin basic protein (MBP) was from Upstate Biotechnology; ML-9 was from Biomol Research Laboratories, Plymouth Meeting, PA; bisindolylmaleimide, Y27632, pertussis toxin, cAMP; Cl*ostridium botulinum* C3 exoenzyme, IKK-2 Inhibitor IV (IKK IV), MG-132, myristoylated PKI and U73122 were from Calbiochem, La Jolla, CA; RNAqueous™ kit was from Ambion, Austin, TX; Effectene Transfection Reagent, QIAEX®II Gel extraction Kit and QIAprep®Spin Miniprep Kit were from Germantown, MD; PCR reagents were from Applied Biosystems, Roche; SuperScript™ II Reverse Transcriptese and TOPO TA Cloning® Kit Dual Promoter were from Invitrogen, Grand Island, NY; EcoR I was from New England Bio Labs, Ipswich, MA; All other chemicals were from Sigma, St. Louis, MO.

New Zealand white rabbits (weight: 4–5 lbs) were purchased from RSI Biotechnology Clemmons, NC and all procedures were conducted in accordance with the Institutional Animal Care and Use Committee of the Virginia Commonwealth University.

### Preparation of dispersed gastric smooth muscle cells

The antrum was separated from the rest of the stomach and the mucosal layer was removed by sharp dissection. Smooth muscle cells from the circular muscle layer of the antrum were isolated by sequential enzymatic digestion of muscle strips, filtration, and centrifugation as described previously [17], [18]. The antrum was cut into thin slices using a Stadie-Riggs tissue slicer and then the slices were incubated for 30 min in a smooth muscle buffer (NaCl 120 mM, KCl 4 mM, KH_2_PO_4_ 2.6 mM, CaCl_2_ 2.0 mM, MgCl_2_ 0.6 mM, HEPES (N-2-hydroxyethylpiperazine-N’ 2-ethanesulfonic acid) 25 mM, glucose 14 mM, and essential amino acid mixture 2.1% (pH7.4)) at 31°C containing 0.1% collagenase (300 U/ml) and 0.01% soybean trypsin inhibitor (w/v). The partly digested tissues were washed twice with 50-ml of collagenase-free smooth muscle buffer and the muscle cells were allowed to disperse spontaneously for 30 min in collagenase-free medium. Cells were harvested by filtration through 500 µm Nitex and centrifuged twice at 350 *g* for 10 min to eliminate broken cells and organelles.

Dispersed muscle cells isolated from the antrum were resuspended in DMEM containing penicillin (200 U/ml), streptomycin (200 µg/ml), gentamycin (100 µg/ml), amphotericin B (2.5 µg/ml) and 10% fetal bovine serum (DMEM-10). The muscle cells were plated at a concentration of 5×10^5^ cells/ml and incubated at 37°C in a CO_2_ incubator. DMEM-10 medium was replaced every three days for 2–3 weeks until confluence was attained. All experiments were done on cells in the first passage [19].

### Transfection of dominant negative mutants, minigene constructs and siRNA into cultured smooth muscle cells

Wild type RGS4, dominant negative RGS4 (RGS4[N88S]), IKK2 (IKK2[K44A]), IkB (IkBa[S32A/S36A]), and phosphorylation-site deficient RhoA (RhoA[S188A]) were subcloned into the multiple cloning site (EcoR I) of the eukaryotic expression vector pcDNA3. Recombinant plasmid DNAs were transiently transfected into the muscle in primary culture using Effectene Transfection Reagent (QIAGEN) for 48 h. Cells were co-transfected with 2 µg of pcDNA3 vector and 1 µg of pGreen Lantern-1 DNA. Transfection efficiency was monitored by the expression of the green fluorescent protein using FITC filters. Control cells were transfected with vector alone [19], [20]. Analysis by fluorescence microscopy showed that approximately 80% of the cells were transfected.

The cDNA sequences encoding the last COOH-terminal 11 amino acids of mouse Ga_q_, Ga_12_, and Ga_13_, and human Ga_i_ were amplified by PCR and verified by DNA sequencing as previously described [21]–[23]. The 5′-end of sense primers contained a BamHI site followed by the ribosome binding consensus sequence (5′-GCCGCCACC-3′), a methionine (ATG) start code, and a glycine (GGA) to protect the ribosome binding site during translation and the nascent peptide against proteolytic degradation. An EcoRI site was synthesized at the 5′-end of the antisense primers immediately after the stop codon (TGA). The purified PCR products were subcloned into the mammalian expression vector pcDNA3.1(+). The oligonucleotide sequence corresponding to the COOH-terminal 11 amino acid residuals of Ga_i_ in random order was synthesized and ligated into pcDNA3.1(+) as a control minigene. All Ga minigene constructs used for transfection experiments were purified with an endotoxin-free maxiprep kit (Qiagen) following the manufacturing protocol.

The siRNAs (small interfering RNA) for PAR-2, Ga_q/11_ and Ga_13_ with lowest predicted off-target potential and 100% homology with the conserved sequences based on human, rat and mouse sequences were selected and obtained from Life Technologies (Grand Island, NY). PAR-2 (ID#s453494): sense: 5′ CCUCCUCUCUGUCAUCUGGTT 3′, antisense: 5′ CCAGAUGACAGAGAGGAGGTC3′; Ga_q/11_ (ID# s5862): sense: 5′ GCAUCAUCGAGUACCCUUUTT 3′, antisense: 5′ AAAGGGUACUCGAUGAUGCCG 3′; and Gα13 (ID#20990): sense: 5′ GCAACGUGAUCAAAGGUAUTT 3′, antisense: 5′ AUACCUUUGAUCACGUUGCTG3′. siRNA transfection efficiency assays were performed with varying concentrations of siRNA (5–50 nM) and it was determined that 40 nM siRNA provided optimal transfection efficiency (data not shown). Non-specific siRNAs were used to determine the efficiency of siRNA. The siRNA transfection was performed using siRNA transfection reagent and siRNA transfection medium according to the manufacturer's instructions.

### Identification of G proteins coupled to PAR2 receptor

G proteins selectively activated by PAR2-AP were identified from the increase in Ga binding to the [^35^S]GTPγS (5′-O-3-thiotriphosphate) using the method of Okamoto et al as described previously [21], [24]. Ten ml of muscle cell suspension (3×10^6^ cells/ml) were homogenized in 20 mM HPES medium (pH 7.4) containing 2 mM MgCl_2_, 1 mM EDTA and 2 mM DTT. After centrifugation at 30,000 g for 15 min, the crude membranes were solubilized for 60 min at 4 °C in 20 mM HEPES medium (pH 7.4) containing 2 mM EDTA, 240 mM NaCl, 0.5% CHAPS (3-[(3-cholamidopropyl) dimethylammonio]-1-pro-panesulfonate), 2 mM PMSF, 20 µg/ml aprotinin, and 20 µM leupetin. The membranes were incubated for 20 min at 37°C with 60 nM [^35^S]GTPgS in the presence or absence of PAR2-AP in a solution containing 10 mM HEPES (pH 7.4), 100 µM EDTA and 10 mM MgCl_2_. The reaction was terminated with 10 volumes of 100 mM of Tris-HCl medium (pH 8.0) containing 10 mM MgCl_2_, 10 mM NaCl and 10 µM GTP, and the mixture was placed in wells precoated with specific antibodies to Ga_q_, Ga_i1_, Ga_i2_, Ga_i3_, Ga_12_, Ga_13_, and Ga_s_. Coating with G protein antibodies (1∶1000) was done after the wells were first coated with anti-rabbit IgG (1∶1000) for 2 h on ice. After incubation for 2 h on ice, the wells were washed three times with phosphate buffer saline solution (PBS) containing 0.05% Tween-20 and the radioactivity from each well was counted by liquid scintillation. The amount of [^35^S]GTPgS bound to the activated Ga subunit was expressed as counts per minute (cpm) per milligram of protein.

### Assay for Phosphoinositide (PI) hydrolysis

Total inositol phosphates were measured by anion exchange chromatography using the method of Berridge et al [25] as described previously [19]. Ten ml of cell suspension (2×10^6^ cells/ml) were labelled with myo-[^3^H] inositol (15 µCi/ml) for 3 h at 31°C. Then cells were centrifuged at 350 g for 10 min to move excess [^3^H]inositol and resuspended in 10 ml of fresh medium. PAR2-AP was added to 0.5 ml of cell suspension and the mixture was incubated in a shaking water bath for 1 min. Cultured smooth muscle cells were labelled with [^3^H]myo-inositols (0.5 µCi/ml) for 24 h in inositol-free DMEM medium. The cultures were washed with phosphate-buffered saline (PBS) and treated with PAR2-AP for 1 min in HEPES medium (pH 7.4). The reaction was terminated by the addition of chloform:methanol:HCl (50∶100∶1 v/v/v). After chloroform (340 µl) and water (340 µl) were added, the samples were vortexed and the phase was separated by centrifugation at 1000 g for 15 min. The upper aqueous phase was applied to a column containing 1 ml of 1∶1 slurry of Dowex AG-1 X8 resin (100–200 mesh in formate form) and distilled water. Total inositol phosphates were eluted with 6 ml of 0.8 M ammonium formate-0.1 M formic acid. The eluates were collected into scintillation vials and counted in gel phase after addition of 10 ml of scintillant. The results were expressed as counts per minute per mg protein.

### Assay for Rho Kinase activity

Rho kinase activity was measured by an immunokinase assay as previously described [21]. Cultured cells were washed one time with PBS, and then were lysed with lysis buffer containing 50 mM Tris-HCl (pH 7.5),150 mM NaCl, 0.1% SDS, 0.5% sodium deoxycholate, 1% NP-40, 10 mM sodium pyrophosphate, and protease inhibitor cocktail (2 µl/ml, BD Biosciences). The homogenates were centrifuged at 10,000 rpm for 10 min at 4 °C. The supernatant containing cytosolic protein was transferred to a fresh tube and 5 µl of Rho kinase antibody was added to each tube and incubated for 2 h at 4 °C followed by overnight incubation at 4 °C with Protein A/G. The pellets were re-suspended in 50 µl of kinase buffer containing 100 mM Tris-HCl (pH 7.4), 1 M KCl, 50 mM MgCl_2_, 10 mM EDTA, and 1 mM DTT. Twenty microliters of Rho kinase immunoprecipitates were added to the reaction mixture containing 100 mM Tris-HCl (pH 7.4), 1 M KCl, 50 mM MgCl_2_, 1 mM DTT, 1 mM ATP, and 10 µCi of [g-^32^P]ATP (3,000 Ci/mol) along with 5 µg of myelin basic protein, followed by incubation for 15 min at 37 °C. Phosphorylation of myelin basic protein was absorbed onto phosphocellulose disks, and free radioactivity was removed by washing 3 times with 75 mM H_3_PO_4_. The amount of radioactivity on the disks was measured by liquid scintillation. The results are expressed as counts per milligram protein per minute.

### Assay for adenylyl cyclase activity

Adenylyl cyclase activity was measured by the formation of cAMP in response to agonists by radioimmunoassay using [^125^I]cAMP [18], [21]. One ml (3×10^6^ cells/ml) of cell suspension was treated for 60 s with forskolin (10 µM) in the presence of 100 µM isobutylmethyl xanthine, either alone or in combination with PAR2-AP (1 µM). The reaction was terminated with cold 6% trichloroacetic acid (v/v) and vortexed vigorously. After centrifugation, the supernatants were extracted three times with water-saturated diethyl ether to remove the tricholoroacetic acid and the samples were then lyophilized and frozen at –20°C. The samples were reconstituted for radioimmunoassay in 50 µl of 50 mM sodium acetate (pH 6.2) and acetylated with triethylamine/acetic anhydride (2∶1 v/v) for 30 min. Cyclic AMP was measured in duplicates using 100 µl aliquots and the results were computed from a standard curve using the Prizm@. The results are expressed as pmol of cAMP/mg protein.

### Phosphorylation of RhoA

Protein phosphorylation was determined from the amount of ^32^P incorporated into each protein after immunoprecipitation with specific antibody to RhoA as previously described [26]. Freshly dispersed cells were incubated with [^32^P] orthophosphate for 4 h. One milliliter of samples was incubated with PAR2-AP (1 µM) for 10 min and the reaction was terminated by rapid centrifugation. The pellet was homogenized in lysis buffer containing 50 mM Tris-HCl (pH 7.5),150 mM NaCl, 0.1% SDS, 0.5% sodium deoxycholate, 1% NP-40, 10 mM sodium pyrophosphate, and protease inhibitor cocktail (2 µl/ml). Cell lysates were separated by centrifugation at 13,000 g for 10 min at 4 °C, precleared with 40 µl of protein A-Sepharose, and incubated with RhoA antibody for 2 h at 4 °C and with 40 µl of protein A-Sepharose for another one hour. The immunoprecipitates were extracted with Laemmli sample buffer, boiled for 5 min, and separated by electrophoresis on SDS-PAGE. After transfer to nitrocellulose membranes [^32^P]RhoA was visualized by autoradiography.

### Western blot analysis

Phosphorylation of MLC_20_, MYPT1, CPI-17, IKK2, and the p65 subunit of NF-kB was measured using phospho-specific antibodies and the degradation of IkB was measured using IkB antibody (1∶1000) as described previously [19]. One milliliter of cell suspension (2×10^6^ cell/ml) was treated with PAR2-AP (1 µM) and solubilized on ice for one hour in medium containing 20 mM Tri-HCl (pH 8.0), 1 mM DTT, 100 mM NaCl, 0.5% sodium dodecyl sulfate, 0.75% deoxycholate, 1 mM PMSF, 10 µg/ml of leupeptin and 100 µg/ml of aprotinin. The proteins were resolved by SDS/PAGE and electrophoretically transferred onto nitrocellulose membranes. The membranes were incubated for 12 h with phospho-specific antibodies to MLC_20_ (Ser^19^), MYPT1 (Thr^696^), CPI-17 (Thr^38^), IKK2(ser^177/181^), p65 subunit (Ser^536^), IkBa or NF-kB, and then for 1 h with horse-radish peroxidase-conjugated secondary antibody (1∶2000). The protein bands were identified by enhanced chemiluminescence reagent.

### Measurement of contraction in dispersed smooth muscle cells

Contraction in freshly dispersed gastric circular smooth muscle cells was determined by scanning micrometry as previously described [19], [27]. An aliquot (0.4 ml) of cells containing approximately 10^4^ cells/ml was treated with 100 µl of medium containing various concentrations of PAR2-AP for different time periods (30 s to 10 min) and the reaction was terminated with 1% acrolein at a final concentration of 0.1%. The composition of the medium was 120 mM NaCl, 4 mM KCl, 2.6 mM KH_2_PO_4_, 0.6 mM MgCl_2_, 25 mM HEPES, 14 mM glucose, 2 mM CaCl_2_, and 2.1% Eagle's essential amino acid mixture. Concentration-response curves for PAR2-AP were constructed for the peak contraction, which occurred at 30 s after addition of PAR2-AP. The mean lengths of 50 muscle cells treated with PAR2-AP were measured by scanning micrometry and compared with the mean lengths of untreated cells. The contractile response was expressed as the percent decrease in mean cell length from control cell length.

### Statistical analysis

The results were expressed as means ± S.E. of *n* experiments. *P* values were determined by an unpaired, two-tailed Student’s *t* test when comparing two samples, by one-way ANOVA with a Dunnett post hoc test when comparing more than two samples to a control, or by one-way ANOVA with a Tukey post hoc test when comparing multiple samples using GraphPad InStat software (Version 3.06 for Windows, San Diego, CA, USA). Each experiment was done on cells obtained from different animals. A probability of p<0.05 was considered significant.

## Results

### Expression of PAR2 and G protein activation by PAR2-AP in smooth muscle

**Figure 1:**
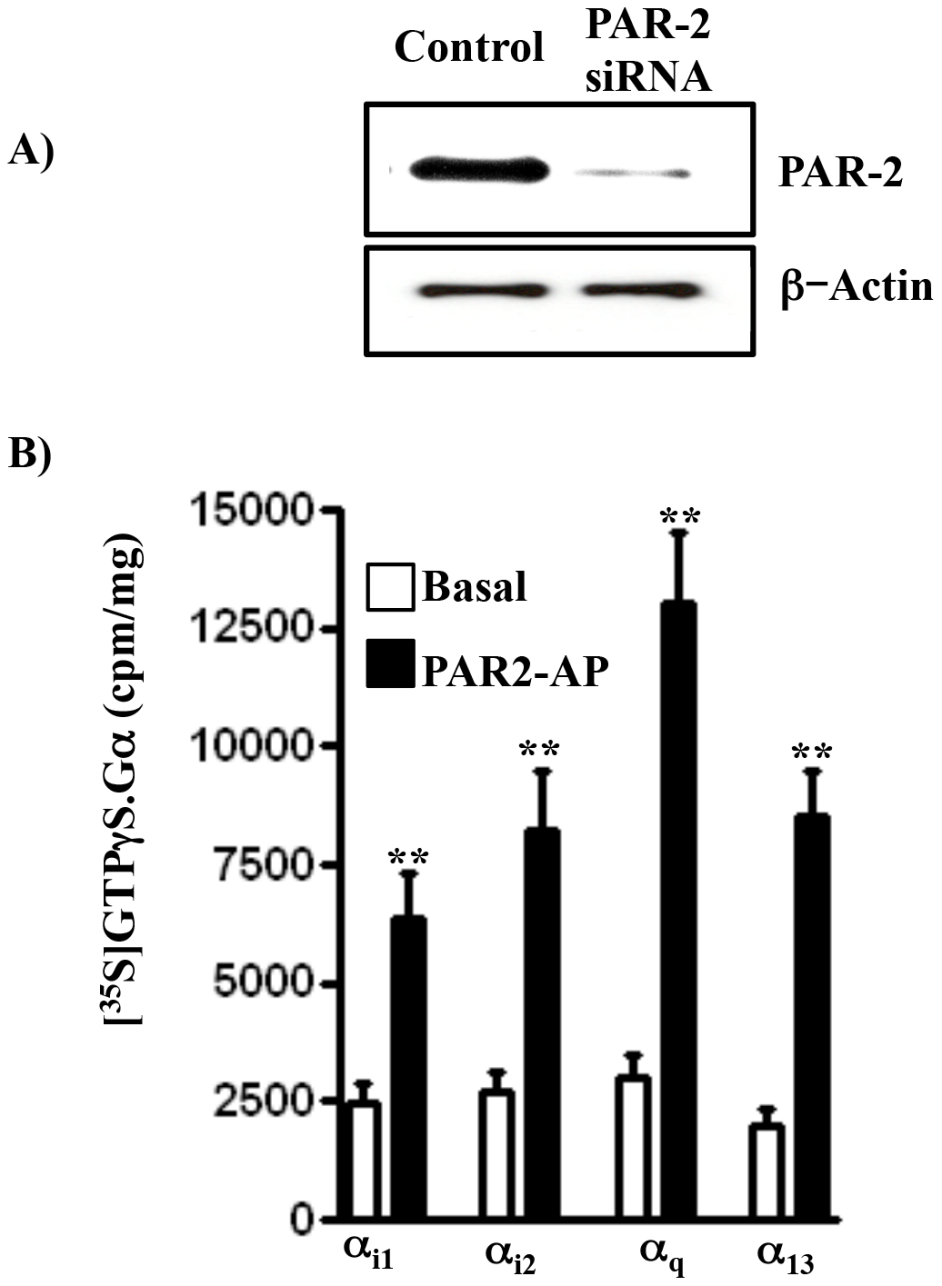
(**A**) Expression of PAR2 in rabbit gastric smooth muscle cells. Western blot was performed on homogenates prepared from cultured gastric smooth muscle cells from rabbit. Lysates were prepared from control cells and cells transfected with PAR2-specific siRNA and proteins were resolved by SDS-polyacrylamide gel electrophoresis, transferred to nitrocellulose membrane, and probed with specific antibodies to PAR2 (1∶1000). Immunoreactive bands for PAR2 was detected by enhanced chemiluminescence. Suppression of PAR2 expression in cells transfected with PAR2 siRNA suggests that selectivity of antibodies and validity of siRNA transfection. (**B**) Activation of G proteins PAR2-AP in gastric smooth muscle cells. Membranes isolated from freshly dispersed smooth muscle cells were incubated with ^35^S-labeled guanosine 5-O-(3-thiotriphosphate) ([^35^S]GTPgS) in the presence or absence of PAR2-activating peptide (PAR2-AP) (1 µM) for 20 min. Aliquots were added to wells coated with Ga_q_, Ga_i1_, Ga_i2_, Ga_i3_, Ga_s_, Ga_12_ or Ga_13_ for 2 h and bound radioactivity was measured. Results are expressed as cpm/mg protein. PAR2-AP caused a significant increase in the binding of [^35^S]-GTPgS-Ga complexes to wells coated with Ga_i1_ and Ga_i2,_ Ga_q_, and Ga_13_. PAR2-AP did not cause significant increase in the binding to Ga_i3_, Ga_12_, and Ga_s_ (7±10% to 11±8% increase above basal levels). Values are means ± S.E. of four experiments. ** Significant increase in G protein activation (P<0.001).

Western blot analysis of homogenates derived from rabbit gastric smooth muscle cells using antibodies to PAR2 (1∶1000 dilution) (Santa Cruz, CA; sc-8206) demonstrated presence of PAR2 in smooth muscle (Fig. 1A). The specificity of antibody was determined using immunizing peptide (Santa Cruz, CA; sc-8206 p) blocking experiment (data not shown) and validated by PAR2 siRNA experiment. Transfection of cells with PAR2-specific siRNA for 48 h greatly suppressed the expression of PAR2 (Fig. 1A).

Studies in various tissues and cell lines suggest that PAR2 are coupled to both pertussis toxin (PTx)-sensitive (G_i_) and -insensitive (G_q_, G_12_/G_13_) G proteins, but the specific G_i_ isoforms and G_12_ and G_13_ proteins coupled to PAR2 have not been identified [4], [7]. Addition of PAR2-AP caused a 2.3-, 3.1-, 4.8-, and 3.2-fold (P<0.01) increase in the binding of Ga_i1_, Ga_i2_, Ga_q_, and Ga_13_, respectively (Fig. 1B). PAR2-AP did not cause significant increase in the binding to Ga_i3_, Ga_12_, and Ga_s_ (7±10% to 11±8% increase above basal levels). These results suggest that PAR2 is coupled to G_i1_, G_i2_, G_q_ and G_13_.

### Stimulation of phosphoinositide (PI)-specific PLC activity (PI hydrolysis) by PAR2-AP

**Figure 2:**
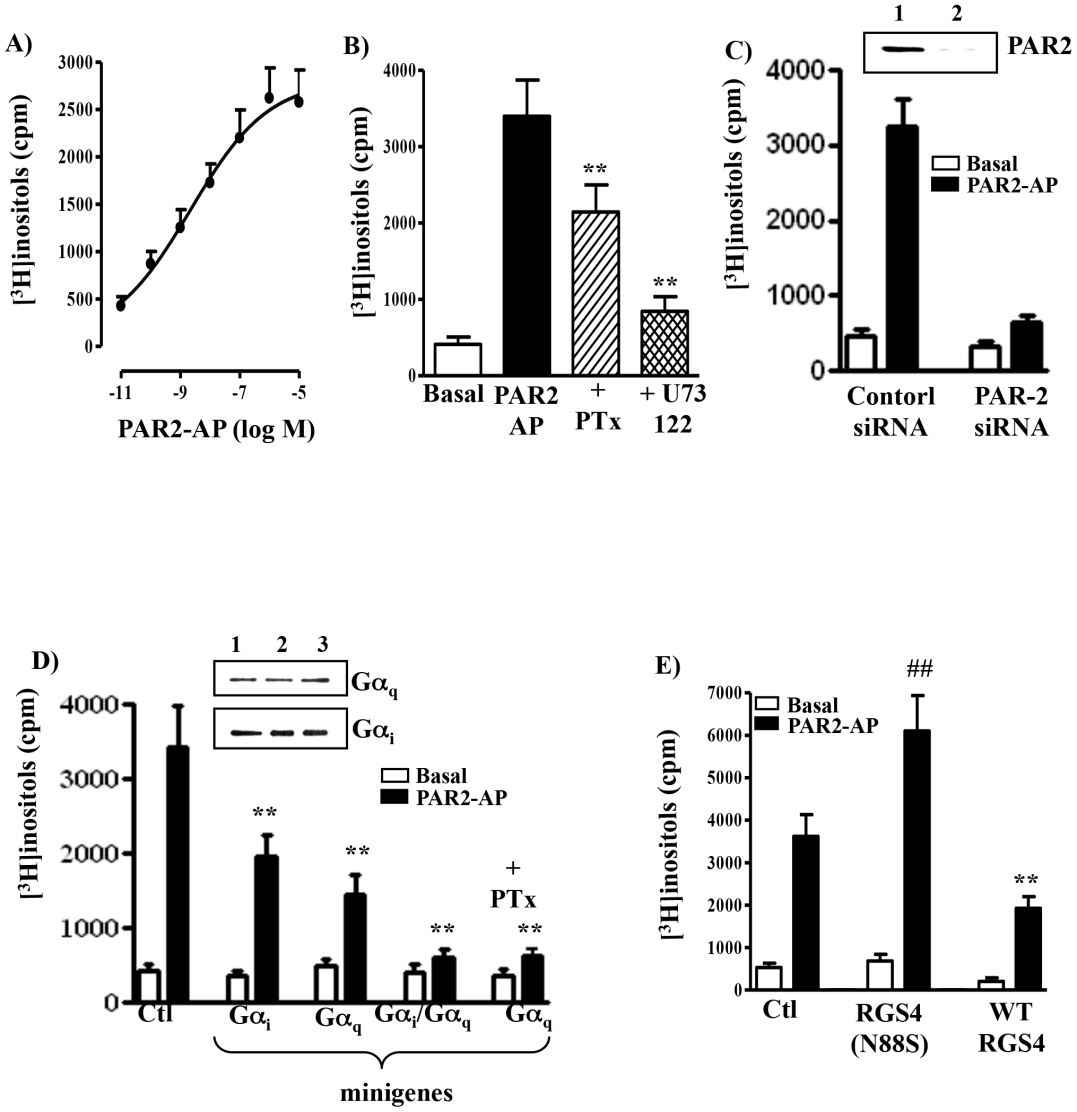
Stimulation of PI hydrolysis by PAR2-AP via Gα_i_ and Gα_q_ proteins. (**A**) Concentration-dependent stimulation of PI hydrolysis PAR2-AP. Freshly dispersed muscle cells labelled with myo-2-[^3^H]inositol were incubated with different concentrations of PAR2-AP for 60 s. Total [^3^H]inositol phosphates were separated by ion-exchange chromatography. Results are expressed as total [^3^H]inositol phosphate formation in cpm/mg protein above basal levels (basal levels: 520±66 cpm/mg protein). (**B**) Selective inhibition of PAR2-AP-stimulated PI hydrolysis by pertussis toxin (PTx). Dispersed muscle cells labelled with myo-2-[^3^H]inositol were incubated for 10 min with U73122 (10 µM) or with PTx (200 ng/ml) for 60 min, and then PAR2-AP (1 µM) for 60 s. Treatment of cells with PTx (563±102 cpm/mg protein) or U73122 (485±86 cpm/mg protein) alone had no effect on basal PI hydrolysis. (**C**) Inhibition of PAR2-AP-stimulated PI hydrolysis by PAR2 siRNA. Control muscle cells and cells transfected with PAR2-specific siRNA were labelled with myo-2-[^3^H]inositol and then treated with PAR2-AP (1 µM) for 60 s. Inset: expression of PAR2 in control cells (lane 1) and cells transfected with PAR2 siRNA (lane 2) was measured by western blot using antibody to PAR2 (1∶1000). (**D**) Inhibition of PAR2-AP-stimulated PI hydrolysis by Ga_q_ minigene, Ga_i_ minigene or both. Cultured gastric muscle cells labelled with myo-2-[^3^H]inositol and expressing Ga_q_ minigene, Ga_i_ minigene, both Gα_q_ and Gα_i_ minigenes, or control vector were treated with PAR2-AP (1 µM) for 60 s. In some experiments cells transfected with Ga_q_ minigene were treated with PTx (200 ng/ml) for 60 min. Inset: expression of Ga_q_ and Ga_i_ in control cells (lane 1), cells transfected with Ga_q_ minigene (lane 2) or Ga_i_ minigene (lane 3) was measured by western blot using antibody to Ga_q_ (1: 1000) or a common antibody to Ga_i_ (1:2000). (**E**) Regulation of PAR2-AP-stimulated PI hydrolysis by regulator of G protein signaling 4 (RGS4). Cultured gastric muscle cells labelled with myo-2-[^3^H]inositol and overexpressing wild type RGS4, dominant negative RGS4 (RGS4[N88S]), or vector alone were treated with PAR2-AP (1 µM) for 60 s. Results are expressed as total [^3^H]inositol phosphate formation in cpm/mg protein. Values are means ± S.E. of four experiments. ** Significant inhibition from control response to PAR2-AP (P<0.001); ## significant increase from control response to PAR2-AP (P<0.01).

PAR2-AP stimulated PI hydrolysis in a concentration-dependent fashion with an EC_50_ of 2 nM and maximal increase of 423±46% (P<0.001) above basal levels (basal activity 520±66 cpm/mg protein) (Fig. 2A). Treatment of cells with pertussis toxin (PTx, 200 ng/ml) for 1 h partially inhibited (33±5%% inhibition, P<0.01) PI hydrolysis in response to PAR2-AP suggesting that PAR2 stimulated PI hydrolysis was mediated by both PTx-sensitive (G_i_) and -insensitive (G_q_) G proteins (Fig. 2B). Stimulation of PI hydrolysis in response to PAR2-AP was abolished by the selective PI hydrolysis inhibitor, U73122 (10 µM) (Fig. 2B). Treatment of cells with PTx alone (563±102 cpm/mg protein) or U73122 alone (485±86 cpm/mg protein) had no effect on basal PI hydrolysis.

Treatment of cells with trypsin (1 µM), which activates PAR2 also stimulated PI hydrolysis (2653±302 cpm/mg protein above basal levels of 439±68 cpm/mg protein) similar to that obtained with PAR2-AP. Evidence for the involvement of PAR2 receptors in the stimulation of PI hydrolysis in response to PAR2-AP was obtained using PAR2-specific siRNA. Increase in PI hydrolysis in response to PAR2-AP was significantly inhibited (83±6%, p<0.001) in cells transfected with PAR2 siRNA (Fig. 2C).

The involvement of G_q_ and G_i_ in mediating PI hydrolysis in response to PAR2-AP was corroborated in experiments using Ga minigenes in cultured smooth muscle cells. Expression of Ga minigenes was confirmed by RT-PCR using primers corresponding to Ga insert and vector. The primers for the Ga minigenes amplified a PCR product of 434 bp when Ga insert are present (data not shown). The synthetic peptide corresponding to the COOH terminus of Ga subunits selectively antagonized G protein activation by blocking receptor-G protein interaction [21]. Previous studies in smooth muscle have shown that expression of minigene plasmid constructs that encode COOH-terminal peptide sequence of Ga_i_ and Ga_q_ selectively blocked G_i_ and G_q_ activation, respectively [21]-[23]. A minigene containing Ga in random order was expressed as a control. Transfection of Ga minigenes had no effect on the expression of Ga_q_ and Ga_i_ levels (Fig. 2D). PAR2-AP (1 µM) induced PI hydrolysis (∼ 5-fold) in cultured smooth muscle cells was closely similar to stimulated PI hydrolysis in freshly dispersed smooth muscle cells. Expression of Ga_q_ minigene or Ga_i_ minigene partially inhibited PI hydrolysis in response to PAR2-AP (62±5% inhibition and 45±4% inhibition, respectively, P<0.01) (Fig. 2D). Co-expression of Ga_q_ and Ga_i_ minigenes or treatment of cells expressing Ga_q_ minigene with PTx additively inhibited PI hydrolysis in response to PAR2-AP (93±6% and 90±7% inhibition, respectively) (Fig. 2D). It is worth noting that the inhibition of PAR2-AP-induced PI hydrolysis in cultured smooth muscle cells expressing Ga_i_ minigene was closely similar to inhibition of PI hydrolysis by PTx in freshly dispersed muscle cells. Expression of control minigene (random minigene) had no effect on PI hydrolysis in response to PAR2-AP (2762±568 above basal levels of 456±76 cpm/mg protein). Transfection of cultured muscle cells with Ga_q_ siRNA also partially inhibited PAR2-AP stimulated PI hydrolysis (1539±203 cpm/mg protein above basal levels of 348±62 cpm/mg protein; 53±4% inhibition compared to control response). Treatment of cells transfected with Ga_q_ siRNA and PTx blocked PI hydrolysis (536±78 cpm/mg protein above basal levels of 382±65 cpm/mg protein). The results corroborate that PI hydrolysis by PAR2 was mediated by both G_q_ and G_i_.

Further evidence for the involvement of G_q_ proteins in the regulation of PI hydrolysis by PAR2-AP was obtained in cultured muscle cells. The strength and duration of Ga-GTP signaling are regulated by a family of GTPase-activating proteins known as regulators of G protein signaling (RGS). Previous studies have shown that Ga_q_-GTPase activity is regulated by RGS4 in smooth muscle cells [20]. To examine whether the activation of PI hydrolysis by PAR2-AP is regulated by RGS4, we overexpressed dominant negative RGS4(N88S) that lacks the ability to stimulate Ga-GTPase activity in cultured muscle cells. PI hydrolysis in response to PAR2-AP was significantly augmented (75±5% increase) in cells overexpressing RGS4(N88S) (Fig. 2E). In contrast, PI hydrolysis in response to PAR2-AP was significantly attenuated (42±4% inhibition) in cells overexpressing wild type RGS4 (Fig. 2E). The pattern implied that PAR2-induced PI hydrolysis was mediated by Ga_q_-dependent activation of PLC-b1 and augmented by inactivation of RGS4.

### Inhibition of adenylyl cyclase by PAR2-AP

**Figure 3:**
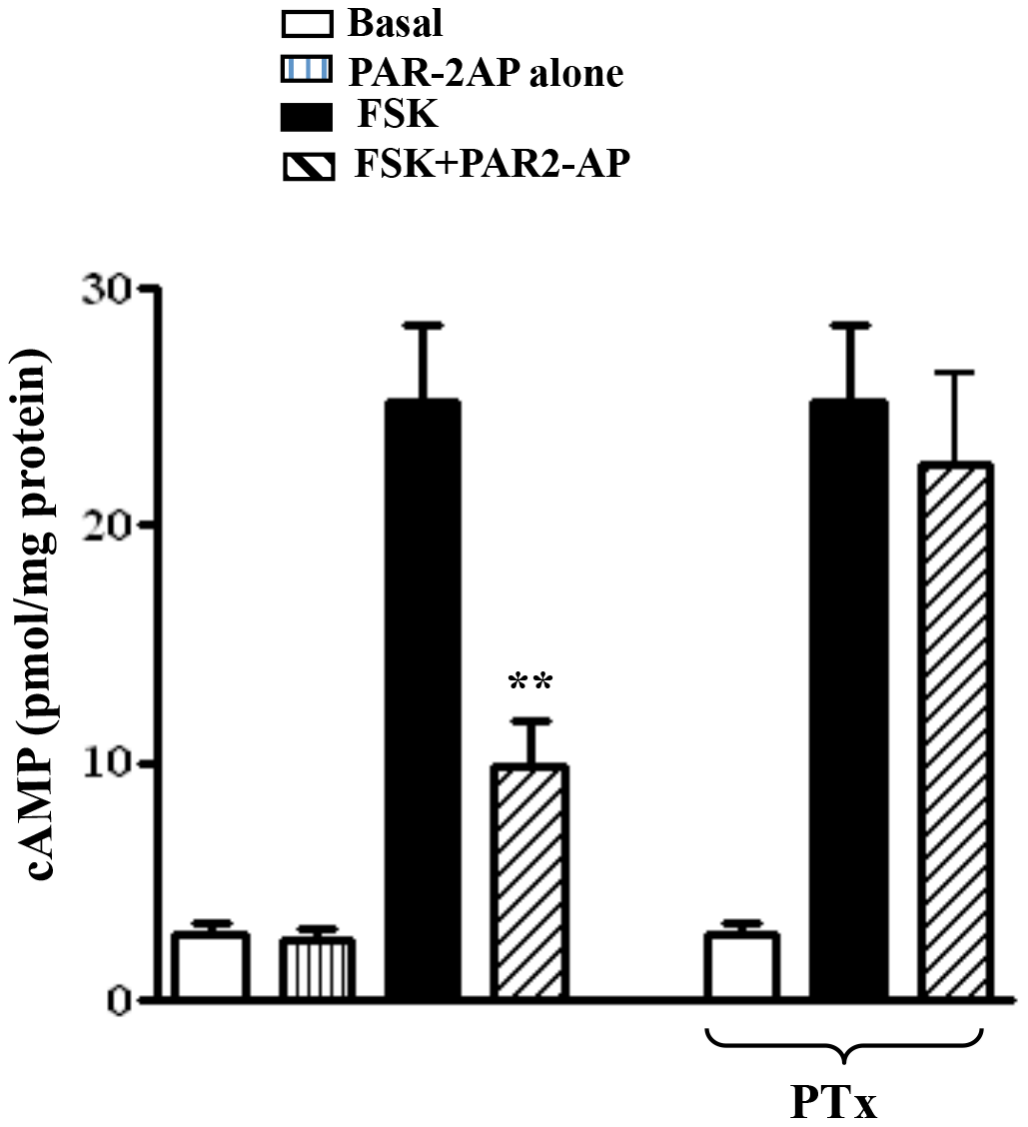
Inhibition of forskolin-stimulated cAMP formation by PAR2-AP. Cyclic AMP formation was measured in freshly dispersed muscle cells in the presence of 100 µM isobutylmethylxanthine. Muscle cells were treated with PAR2-AP (1 µM) alone, forskolin (FSK, 10 µM) alone or forskolin plus PAR2-AP (1 µM) for 60 s and cAMP was measured by radioimmunoassay. In some experiments cells were treated with pertussis toxin (PTx, 200 ng/ml) for 60 min, and then treated with forskolin (10 µM) and PAR2-AP (1 µM) for 60 s. Results are expressed as pmol/mg protein. Values are means ± S.E. of 4 experiments. ** Significant inhibition of forskolin-stimulated cAMP (P<0.001).

Freshly dispersed smooth muscle cells were used to examine the ability of G_i_-coupled PAR2 to inhibit forskolin-stimulated cAMP formation. Treatment of muscle cells with forskolin (10 µM) for 10 min significantly increased cAMP formation (22.1±1.86 pmol/mg protein above basal level of 2.8±0.5 pmol/mg protein) in the presence of 100 µM isobutyl methyl xanthine. PAR2-AP (1 µM) inhibited forskolin-stimulated cAMP formation by 61±3% (Fig. 3). Preincubation of muscle cells with PTx (200 ng/ml) for 1 h significantly attenuated the inhibitory effect of PAR2-AP (15±2% inhibition of forskolin stimulated cAMP formation) (Fig. 3). The results are consistent with the activation of G_i2_ and G_i3_ by PAR2-AP. Treatment of cells with PAR2-AP alone had no significant effect on cAMP levels (2.6±0.4 pmol/mg protein in the presence of PAR2-AP versus 2.8±0.5 pmol/mg protein basal levels) (Fig. 3).

### Activation of Rho kinase by PAR2-AP

**Figure 4:**
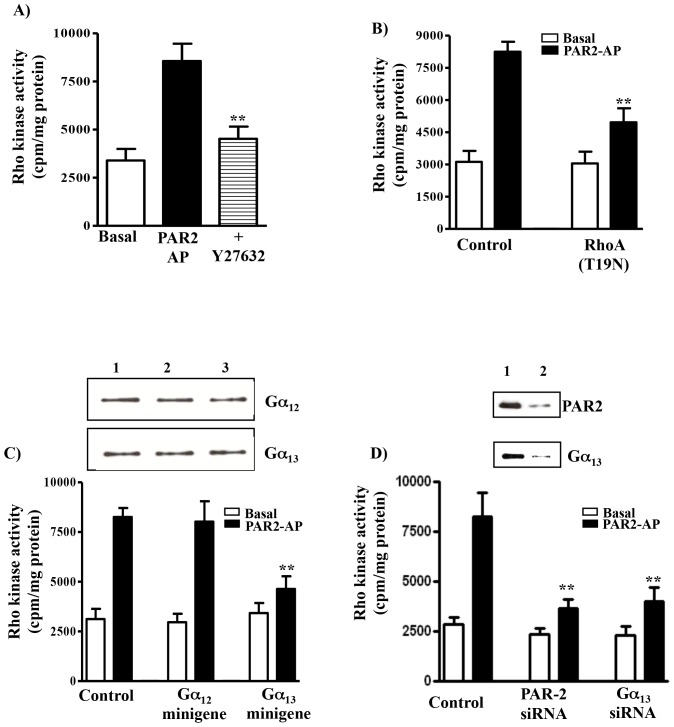
Ga_13_/RhoA-dependent stimulation of Rho kinase activity by PAR2-AP. (**A**) Freshly dispersed muscle cells were incubated with PAR2-AP (1 µM) for 10 min and Rho kinase activity was measured by immunokinase assay. Y27632 was used in vitro in the assay to determine the specificity of Rho kinase activity. (**B**) Inhibition of PAR2-AP-stimulated Rho kinase activity by dominant negative RhoA (RhoA[T19N]). Cultured smooth muscle cells expressing dominant negative RhoA (RhoA[T19N]) or vector alone were incubated with PAR2-AP (1 µM) for 10 min. (**C**) Inhibition of PAR2-AP-stimulated Rho kinase activity by Ga_13_ minigene. Cultured gastric muscle cells expressing Ga_12_ or Ga_13_ minigene, or control vector were treated with PAR2-AP (1 µM) for 10 min. Inset: expression of Ga_12_ and Ga_13_ in control cells (lane 1), cells transfected with Ga_12_ minigene (lane 2) or Ga_13_ minigene (lane 3) was measured by western blot using antibody to Ga_12_ (1∶1000) or Ga_13_ (1∶1000). (**D**) Inhibition of PAR2-AP stimulated Rho kinase activity by PAR2 or Ga_13_ siRNA. Control muscle cells and cells transfected with PAR2 siRNA or Ga_13_ siRNA were treated with PAR2-AP (1 µM) for 10 min. Inset: (top panel) expression of PAR2 in control cells (lane 1) and cells transfected with PAR2 siRNA (lane 2) was measured by western blot using antibody to PAR2 (1:1000); (bottom panel) expression of Gα_13_ in control cells (lane 1) and cells transfected with Ga_13_ siRNA (lane 2) was measured by western blot using antibody to Ga_13_ (1:1000). Rho kinase activity was measured using [^32^P]ATP by immunokinase assay. Results are expressed as cpm/mg protein. Values are means ± S.E. of four experiments. ** Significant inhibition from control response (P<0.001).

In freshly dispersed smooth muscle cells PAR2-AP stimulated Rho kinase activity by 152±12% above basal levels (basal levels: 3363±587 cpm/mg protein) (Fig. 4A). The specificity of the immunokinase activity was determined using a selective inhibitor of Rho kinase (Y27632, 1 µM) (Fig. 4A). Treatment of cells with Y27632 (1 µM) alone had no effect on basal Rho kinase activity (3025±425 cpm/mg protein).

PAR2-AP also stimulated Rho kinase activity in cultured smooth muscle cells (164±13% increase) that was not significantly different from the response in freshly dispersed muscle cells. Overexpression of dominant negative RhoA (RhoA[T19N]) in cultured muscle cells significantly inhibited Rho kinase activity in response to PAR2-AP (Fig. 4B), suggesting activation of Rho kinase is downstream of RhoA. Evidence for the involvement of G_13_ in PAR2 mediated Rho kinase activity was obtained by expression of Ga minigenes in cultured smooth muscle cells. Expression of Ga_12_ or Ga_13_ levels was not altered by transfection of Ga minigenes (Fig. 4C). Expression of the Ga_13_ minigene abolished (64±4% inhibition; P<0.01) Rho kinase activation in response to PAR2-AP, whereas expression of the Ga_12_ minigene had no effect (Fig. 4C), Treatment of smooth muscle cells with PAR1-AP also stimulated Rho kinase activity (121±16% above basal levels of 3425±502 cpm/mg protein). The response to PAR1-AP was blocked by the expression of the Ga_12_ minigene (72±8% inhibition; P<0.01), but not by the expression of the Ga_13_ minigene (8±4% inhibition). These results suggest that PAR2-stimulated Rho kinase activity is mediated via G_13_ and consistent with selective activation of G_13_ but not G_12_ by PAR2-AP.

Further evidence for the involvement of PAR2 and Ga_13_ in the stimulation of Rho kinase activity in response to PAR2-AP was obtained using PAR2-specific and Ga_13_-specific siRNA. Increase in Rho kinase activity in response to PAR2-AP was significantly inhibited in cells transfected with PAR2 siRNA (72±5% inhibition, p<0.001) or Gα_13_ siRNA (63±7% inhibition, p<0.001) (Fig. 4D).

### Contraction induced by PAR2-AP in smooth muscle

**Figure 5:**
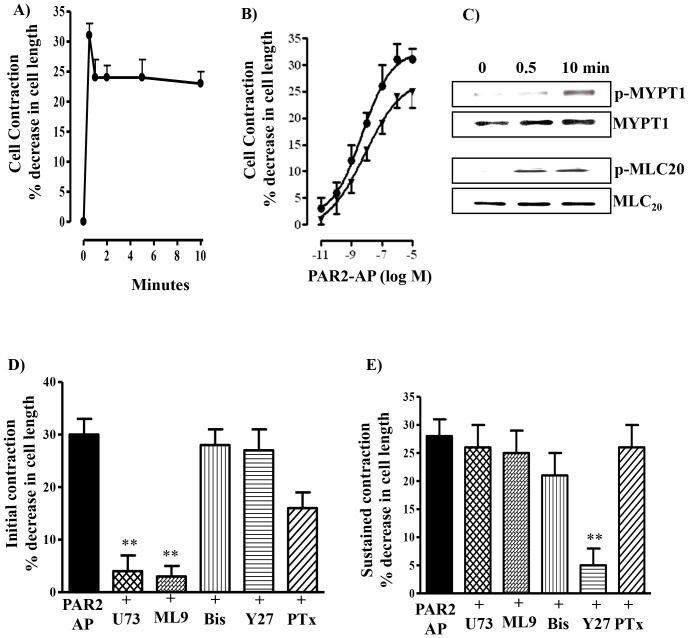
Muscle contraction and phosphorylation of MYPT1 and MLC_20_ in response to PAR2-AP. (**A**) Time course of PAR2-AP-induced contraction in dispersed gastric smooth muscle cells. PAR2-AP (1 µM) was added to freshly isolated muscle cells for different periods of time ranging from 30 s to 10 min. (**B**) Concentration-response curves for the contractile effect of PAR2-AP. PAR2-AP was added to freshly isolated muscle cells at a concentration ranging from 10 pM to 1 µM, and peak contraction at 30 s (solid circles) and sustained contraction at 10 min (solid triangles) were measured. (**C**) Phosphorylation of MYPT1, and MLC_20_ by PAR2-AP in dispersed muscle cells. Freshly dispersed muscle cells were treated with PAR2-AP for 30 s and 10 min. MLC_20_ phosphorylation was measured using phospho-specific-Ser^19^ MLC_20_ antibody. MYPT1 phosphorylation was measured using phospho-specific-Thr^696^ MYPT1 antibody. (**D and E**) Effect of inhibitors on initial and sustained contraction in response to PAR2-AP. Freshly dispersed muscle cells were treated separately with the PLC inhibitor U73122 (10 µM), MLC kinase inhibitor ML-9 (10 µM), PKC inhibitor bisindolylmaleimide (1 µM), Rho kinase inhibitor Y27632 (1 µM) for 10 min, or pertussis toxin (200 ng/ml) for 60 min, and then treated with PAR2-AP (1 µM) for 30 s (initial) or 10 min (sustained). Muscle contraction was measured by scanning micrometry, and the results are expressed as percent decrease in cell length from control cell length (105±3 µm). Values are means ± S.E. of six experiments. ** Significant inhibition from control response (P<0.001).

Consistent with its ability to stimulate PI hydrolysis and Rho kinase activity, PAR2-AP caused contraction of dispersed gastric smooth muscle cells and the contraction was characterized by an initial transient phase followed by a sustained phase (Fig. 5A). Both initial and sustained contraction induced by PAR2-AP was concentration-dependent with an EC_50_ of 1 nM and 6 nM, respectively (Fig. 5B). Maximal initial contraction induced by PAR2-AP (32±3% decrease in cell length from a control cell length of 105±3 µm) was similar to that elicited by other contractile agonists, such as acetylcholine (31±3% decrease in cell length), cholecystokinin (29±2% decrease in cell length) or 5-hydroxytryptamine (30±4% decrease in cell length).

MLC_20_ phosphorylation, measured using phospho-specific antibody (MLC_20_ Ser^19^), by PAR2-AP was rapid (within 30 s) and sustained (10 min) closely paralleling the biphasic nature of contraction (Fig. 5C).

### Pathways mediating contraction induced by PAR2-AP

Initial (30 s) contraction by PAR2-AP was abolished by the PLC-b inhibitor U73122 (10 µM) and the MLC kinase inhibitor ML-9 (10 µM) (Fig. 5D). Pretreatment of muscle cells with PTx (200 ng/ml) for 1 h partly inhibited initial contraction induced by PAR2-AP (Fig. 5D). The partial inhibition of contraction by PTx implied participation of both G_q_ and G_i_ in stimulation of PI hydrolysis and the results are consistent with the PAR2-AP-induced activation of PI hydrolysis by both G_q_ and G_i_ proteins and selective inhibition of PI hydrolysis by PTx. Initial contraction induced by PAR2-AP was not affected by the PKC inhibitor bisindolylmaleimide (1 µM) or the Rho kinase inhibitor Y27632 (1 µM) (Fig. 5D).

Sustained contraction by PAR2-AP, however, was preferentially blocked by the Rho kinase inhibitor Y27632 (1 µM) (Fig. 5E), but not by U73122, ML-9 or bisindolylmaleimide (Fig. 5E). Treatment of cells with PAR2-AP for 10 min also induced MYPT1 phosphorylation at Thr^696^ (Fig. 5C).

### Activation of nuclear factor NF-kB pathway by PAR2-AP

**Figure 6:**
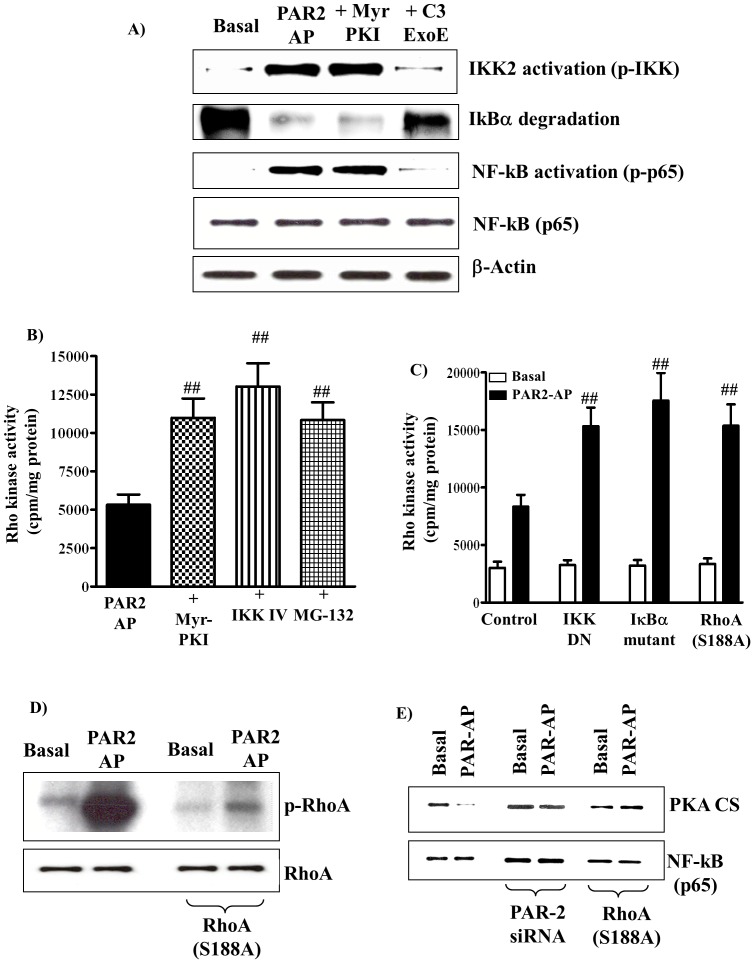
Feedback inhibition of PAR2-AP-stimulated Rho kinase activity by PKA derived from NF-kB pathway. (**A**) Gastric muscle cells were incubated with myristoylated PKI for 10 min or with the RhoA inhibitor *Clostridium botulinum* C3 exoenzyme (2 µg/ml) for 2 h, and then treated with PAR2-AP for 10 min. Cells were homogenized in lysis buffer and proteins were separated on SDS-PAGE. Phosphorylation (i.e., activation) of IKK2 was measured by western blot phospho-specific (Ser^177/181^) antibody and phosphorylation of p65 subunit (activation of NF-kB) was measured using phospho-specific (Ser^536^) antibody. Degradation of IkBa was measured using antibody to IkBa. Western blot of the b-actin protein is shown for a control loading. (**B**) Freshly dispersed gastric smooth muscle cells were treated with PAR2-AP (1 µM) for 10 min in the presence or absence of IKK IV (10 µM), MG-132 (10 µM), or myristoylated PKI (1 µM). Results are expressed as cpm/mg protein above basal levels (3125±502 cpm/mg protein). Treatment of cells with IKK IV (3021±486 cpm/mg protein), MG-132 (2859±602 cpm/mg protein), or myristoylated PKI (3254±574 cpm/mg protein) alone had no effect on basal Rho kinase activity. (**C**) Cultured smooth muscle cells expressing dominant negative mutants of IKK2 (K44A) or IkBa (S32A/S36A) or the PKA phosphorylation site-deficient RhoA (S188A) were treated with PAR2-AP (1 µM). Rho kinase activity was measured using [^32^P]ATP by immunokinase assay. Results are expressed as cpm/mg protein. Values are means ± S.E. of four experiments. ## Significant increase from control response to PAR2-AP (P<0.01). (**D**) Phosphorylation of RhoA was measured in cells labelled with [^32^P]Pi. Control cells or cells expressing phosphorylation-site deficient RhoA(S188A) were treated with PAR2-AP (1 µM) and phosphorylation of RhoA was analysed by SDS-PAGE and autoradiography. (**E**) Dissociation of PKA catalytic subunit from NF-kB. Control cells, cells transfected with PAR2 siRNA or RhoA(S188A) were treated with PAR2-AP for 10 min. NF-kB immunoprecipitates were separated by SDS/PAGE and membranes probed with antibodies to the catalytic subunit of PKA (PKACS, 1∶2000) or NF-kB (1:1000).

NF-kB is activated by a variety of stimuli, cytokines (e.g., IL-1β) and G protein-coupled receptor agonists (e.g. lipopolysacchride). The canonical pathway for activation of NF-kB involves phosphorylation of Ik-Ba by IkBa kinase (IKK2), degradation of IkBa via the proteasomal pathway, and translocation of NF-kB dimer to the nucleus. We have examined whether PAR2s are coupled to activation of NF-kB in smooth muscle cells. Activation of IKK2 was measured by phosphorylation of IKK2 using phospho-specific substrate (Ser^176/180^), IkBa degradation was measured by western blot and NF-kB activation was measured by phosphorylation of p65 subunit using phospho-specific antibody (Ser^536^). PAR2-AP (1 µM) induced activation of IKK2, degradation of IkBa and activation of NF-kB in cultured muscle cells. The effect of PAR2-AP was abolished by treatment of cells with the RhoA inhibitor (C3 exoenzyme (2 µg/ml)) suggesting that activation of NF-kB was downstream of RhoA (Fig. 6A)

### Feedback inhibition of RhoA by G_13_-coupled PAR2

Activation of NF-kB pathway by PAR2-AP via RhoA-dependent pathway, and activation of PKA via activation of NF-kB and regulation of RhoA activity by PKA raised the possibility of feedback regulation of RhoA/Rho kinase pathway by NF-kB-dependent mechanism [26]. PAR2-AP-induced Rho kinase activity was significantly augmented by IKK IV (10 µM), MG-132 (10 µM) or myristoylated PKI (1 µM) (Fig. 6B). Treatment of cells with IKK IV (3021±486 cpm/mg protein)), MG-132 (2859±602 cpm/mg protein), or myristoylated PKI (3254±574 cpm/mg protein) alone had no effect on basal Rho kinase activity (3125±502 cpm/mg protein). The results imply that feedback inhibition of PAR2-induced Rho kinase is mediated by PKA. The results also imply that PKA derived from activation of the canonical NF-kB pathway inhibited RhoA. This notion was examined by measurements of Rho kinase activity in cells expressing PKA phosphorylation-site deficient RhoA (S188A) to block phosphorylation of RhoA by PKA and in cells expressing dominant negative mutants of IKK2 and IkBa to block activation of PKA. Rho kinase activity stimulated by PAR2-AP was augmented in cells expressing RhoA(S188A), IKK2(K44A),and IkBa (S32A/S36A) (Fig. 6C). The results imply that PAR2-induced Rho kinase activity was inhibited in a feedback mechanism via phosphorylation of RhoA at Ser^188^ by PKA and the PKA was derived from activation of NF-kB pathway. In support to this notion treatment of cells with PAR2-AP caused RhoA phosphorylation, measured in cells metabolically labelled with ^32^P, and the effect of PAR2-AP was blocked in cells expressing phosphorylation-deficient RhoA (S188A) (Fig. 6D).

Further evidence for the release of PKA catalytic subunit from NF-kB complex in response to PAR2 activation was obtained in co-immunoprecipitation experiments. In the basal state PKA catalytic subunit was co-immunoprecipated with NF-kB. Treatment of cells with PAR2-AP attenuated the amount of PKA catalytic subunit co-immunoprecipitated with NF-kB suggesting dissociation of the catalytic subunit from NF-kB complex (Fig. 6E). The effect of PAR2-AP on NF-kB and PKA catalytic subunit dissociation was blocked in cells transfected with PAR2-specific siRNA or in cells expressing RhoA(S188A) (Fig. 6E).

## Discussion

The gastrointestinal tract, of all the body systems, is exposed to significant amounts of serine proteases in both normal situations and during inflammatory bowel diseases. However, expression of PAR2 receptors and the mechanism underlying their effects on smooth muscle of the gastrointestinal tract are as yet not completely understood. Insights into the mechanism of the PAR2 effects on smooth muscle are important to understand the underlying mechanism involved in altered muscle contractions related to inflammatory bowel diseases.

**Figure 7:**
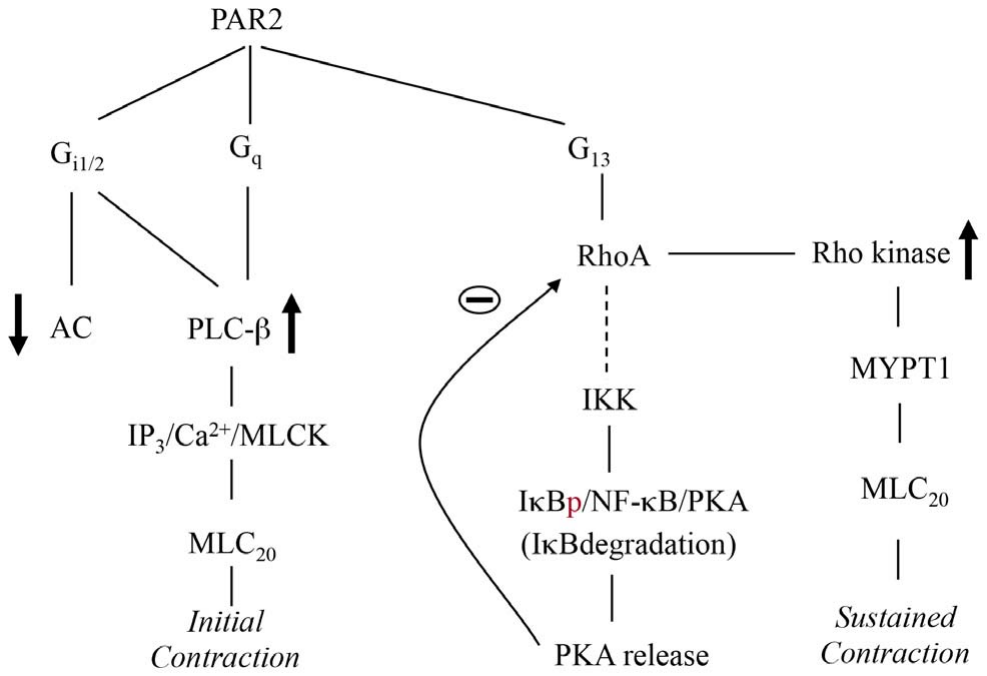
Signaling by PAR2 in smooth muscle. In gastric smooth muscle PAR2 receptors are coupled to G_q_, G_13_ and Gi_1/2_, and stimulation of PI hydrolysis via Ga_q_ and Gbg_i1/2_, and RhoA/Rho kinase via Ga_13_. Initial contraction is mediated via PLC-b/Ca^2+^/MLCK pathway whereas sustained contraction is mediated via RhoA/Rho kinase pathway. Although PAR2 receptors are coupled to inhibition of cAMP via Ga_ i1/2_, activation of NF-kB pathway via Ga_13_/RhoA leads to degradation of IkB and activation of PKA that result in attenuation of Rho kinase activity in a feedback mechanism via phosphorylation of RhoA at Ser^188^ by PKA.

The present study characterized the signaling pathways mediated by PAR2 in gastric smooth muscle cells using biochemical, molecular and functional methods. The results demonstrate the expression of PAR2 in smooth muscle cells and their ability to cause biphasic contraction and MLC_20_ phosphorylation. The initial contraction reflected activation of PLC-β via PTx-sensitive, G_i1_ and G_i2_, and PTx-insensitive G_q_. The sustained contraction reflected activation of RhoA via G_13_ and inhibition of MLC phosphatase via Rho kinase-mediated phosphorylation of the MLC phosphatase regulatory subunit MYPT1 at Thr^696^ [19], [26], [28], [29]. Activation of RhoA also results in the stimulation of the canonical NF-kB pathway and activation of cAMP-independent PKA, which, in turn, phosphorylates RhoA at Ser^188^ and causes feedback inhibition of RhoA activity (Fig. 7).

The expression of PAR2s in smooth muscle cells of the gut is consistent with their expression in other cells types of the gut such as enteric neurons [8], myenteric glia [30], epithelial cells of intestine and colon [31], [32], and endothelial cells and vascular smooth muscle cells of the gut [33], [34]. Studies in various cell lines suggest that PAR2s are coupled to both PTx-sensitive and PTx–insensitive G proteins, but the specific G proteins isoforms coupled to each receptor type have not been characterized [35]. We have demonstrated that in gastric smooth muscle cells, PAR2 receptors are coupled to G_i1_, G_i2_, G_q_ and G_13_. PI hydrolysis stimulated by PAR2 was partially inhibited by suppression of Ga_q_ or by expression of Ga_q_ or Ga_i_ minigene, and additively by co-expression of Ga_q_ and Ga_i_ minigenes suggesting activation of both Ga_q_-dependent PLC-b1 and Gbg_i_-dependent PLC-b3 [36]. In smooth muscle activation of PLC-b1/b3 results in IP_3_-dependent Ca^2+^ release, Ca^2+^/calmodulin-dependent MLC kinase, phosphorylation of MLC_20_ and initiation of muscle contraction [29], [36]. Consistent with this scheme, blockade of IP_3_ generation by PLC-b inhibitor, U73122 or MLC kinase activity by ML-9 inhibited initial contraction by PAR2.

Rho kinase activity stimulated by PAR2 was inhibited by Ga_13_ minigene or Ga_13_ siRNA suggesting activation of Ga_13_-dependent activation of RhoA. Activation of RhoA by Ga_q_, Ga_12_, or Ga_13_ is mediated by various Rho-specific guanine nucleotide exchange factors (RhoGEFs) that promote exchange of GDP for GTP [37]. The RhoGEF family of proteins includes p115RhoGEF, PDZ-RhoGEF, and LARG (*l*eukemia-*a*ssociated *R*ho*G*EF). RhoGEF shares common motifs responsible for binding to activated Ga subunits and for the exchange of GDP for GTP on RhoA [37]. The involvement of different RhoGEFs in the activation of RhoA is receptor-specific. For example, in cell lines PAR1-induced activation of RhoA is mediated by LARG, whereas LPA1-induced activation of RhoA is mediated by PDZ-RhoGEF [38]. In smooth muscle activation of PAR2 results in stimulation of Rho kinase activity and Rho kinase-dependent phosphorylation of MYPT1, a 130-kDa myosin phosphatase targeting subunit that inhibits the MLC phosphatase activity leading to MLC_20_ phosphorylation and muscle contraction. Consistent with this scheme, blockade of Rho kinase activity by Y27632 inhibited sustained contraction in response to PAR2-AP.

Although PAR2 are coupled to inhibition of cAMP formation, activation of PAR2 results in the stimulation of cAMP-independent protein kinase A (PKA) activity via RhoA-dependent mechanism involving activation of canonical NF-kB pathways. The notion that PKA derived from degradation of IkB results in phosphorylation of RhoA at Ser^188^ and inhibition of Rho kinase activity was corroborated using several complimentary approaches. We first measured the activation of canonical NF-kB pathway by PAR2. In the second approach we used IKK2 inhibitor that blocks IkB phosphorylation and subsequent degradation. In the third approach we used a proteasomal inhibitor, MG-132 that prevents degradation of IkB and thus prevents release of PKA catalytic subunits. In the fourth approach we used the membrane-permeable specific PKA inhibitory peptide, myristoylated PKI 14-22 amide. PKI contains PKA pseudosubstrate sequence and specifically inhibits PKA catalytic subunit by binding to the substrate-binding site. In the fifth approach we used a dominant negative mutant IKK2 that lacks the ability to phosphorylate IkB, a prerequisite for degradation. In the sixth approach we used a mutant RhoA (S188A) that lacks the PKA phosphorylation site. In the final approach we measured PAR2-AP-induced dissociation of NF-kB and PKA catalytic subunit by co-immunoprecipitation studies.

We concluded that PAR2-stimulated RhoA was inhibited in a feedback mechanism via cAMP-independent PKA derived from RhoA-dependent activation of canonical NF-kB pathway based on the following evidence: i) PAR2-AP induced phosphorylation (i.e activation) of IKK2, degradation of IkB, and phosphorylation of p65 subunit of NF-kB (activation of NFkB); ii) PAR2-AP-induced IKK2 activation, IkB degradation and NF-kB activation was abolished by a RhoA inhibitor, C3 exoenzyme; iii) PAR2-AP-stimulated Rho kinase activity was significantly augmented by PKI, IKK IV or MG132 in freshly dispersed muscle cells and in cells expressing IKK(K44A), IkBa (S32/S36A) or RhoA(S188A), and iv) PAR2-AP stimulated dissociation of the PKA catalytic subunit from NF-kB complex.

Although PAR2s receptors are coupled to G_i_ and inhibition of cAMP, they stimulated PKA. The PKA holoenzyme is a heterotetramer consisting of two catalytic subunits bound to two regulatory subunits. Classically, the mechanism of PKA activation in response to cAMP elevating agents (G_s_-coupled receptor agonist or forskolin) involves release of the catalytic subunits upon binding of two cAMP molecules to each of the regulatory subunit. Apparently, a certain pool of PKA catalytic subunit is found to be associated with the NF-kB inhibitor protein, IkB. Recent studies have identified novel cAMP-independent mechanism for activation of PKA by various G protein-coupled receptor agonists [39]. The mechanism involves release of PKA catalytic subunit from the IkB complex upon phosphorylation and degradation of IkB. In the basal state, a pool of PKA catalytic subunits is maintained in an inactive state through association with inhibitor of NF-kB (IkB) in an NF-kB/IkB/PKA (catalytic subunits) complex, and this pool of PKA is not sensitive to changes in intracellular cAMP levels. IkB retains the catalytic subunits of PKA in the inactive state, presumably by masking its ATP binding site, and the signals that lead to phosphorylation and degradation of IkB, a prerequisite for NF-kB activation, results in release and activation of the PKA catalytic subunits. The significance of activation of NF-kB in response to PAR2 in smooth muscle requires further research. Smooth muscles respond to inflammatory mediators by synthesizing and secreting various pro- and anti-inflammatory mediators that, in turn, act in autocrine and paracrine fashion to stimulate the expression of other cytokines, chemokines, growth factors, and cell-adhesion molecules [40]-[42]. The expression of some of these mediators and regulation of RGS4 expression by cytokines in smooth muscle are dependent on NF-kB activation [42], [43]. Determination of physiological significance of NF-kB activation by PAR2 may prove significant.

The finding that activation of PKA by a contractile agonist raises an interesting possibility for regulation of signaling molecules involved in the contractile pathway by PKA. It is well established that activation of PKA in gastric smooth muscle cells inhibits muscle contraction, resulting from decrease in [Ca^2+^]_i_ and MLC_20_ phosphorylation. These effects of PKA are a consequence of the inhibitory action of PKA on multiple components of the Ga_q_/Ga_13_ signaling culminating in muscle relaxation.

In conclusion, our studies have demonstrated the expression of PAR2 in smooth muscle and identified the receptor-specific signal transduction pathways in mediating smooth muscle contraction. In gastric smooth muscle cells PAR2 are coupled to activation of G_q_, G_13_, G_i1_, and G_i2_, but not G_i3_, G_12_, G_s_, and stimulation of PI hydrolysis via both Ga_q_ and Gbg_i_. PAR2 are coupled to activation of RhoA/Rho kinase via Ga_13_. PAR2 induced initial contraction was mediated by stimulation of PLC-b activity, generation of IP_3_, IP_3_-dependent Ca^2+^ release, Ca^2+^/calmodulin-dependent activation of MLC kinase and phosphorylation of MLC_20_ at Ser^19^. Whereas sustained contraction was mediated by sequential activation of G_13_, RhoA and Rho kinase, and Rho kinase-dependent phosphorylation of MYPT1 at Thr^696^ and inhibition of MLC phosphatase. Although PAR2s are coupled to inhibition of cAMP, they stimulated cAMP-independent PKA activity via RhoA-dependent canonical NF-kB pathway and this resulted in feedback inhibition of Rho kinase activity via inhibitory phosphorylation of RhoA at Ser^188^.
